# Real-Time Extension of TAO-DFT

**DOI:** 10.3390/molecules28217247

**Published:** 2023-10-24

**Authors:** Hung-Yi Tsai, Jeng-Da Chai

**Affiliations:** 1 Department of Physics, National Taiwan University, Taipei 10617, Taiwan; b04202028@ntu.edu.tw; 2 Center for Theoretical Physics and Center for Quantum Science and Engineering, National Taiwan University, Taipei 10617, Taiwan; 3 Physics Division, National Center for Theoretical Sciences, Taipei 10617, Taiwan

**Keywords:** RT-TAO-DFT, TAO-DFT, multi-reference character, real-time electron dynamics, time-dependent properties, high-order harmonic generation

## Abstract

Thermally assisted occupation density functional theory (TAO-DFT) has been an efficient electronic structure method for studying the ground-state properties of large electronic systems with multi-reference character over the past few years. To explore the time-dependent (TD) properties of electronic systems (e.g., subject to an intense laser pulse), in this work, we propose a real-time (RT) extension of TAO-DFT, denoted as RT-TAO-DFT. Moreover, we employ RT-TAO-DFT to study the high-order harmonic generation (HHG) spectra and related TD properties of molecular hydrogen $H_{2}$ at the equilibrium and stretched geometries, aligned along the polarization of an intense linearly polarized laser pulse. The TD properties obtained with RT-TAO-DFT are compared with those obtained with the widely used time-dependent Kohn–Sham (TDKS) method. In addition, issues related to the possible spin-symmetry breaking effects in the TD properties are discussed.

## 1. Introduction

Over the last thirty years, Kohn–Sham density functional theory (KS-DFT) [1] has been a popular electronic structure method for the ground-state (GS) properties of physical systems in the presence of static external potentials at zero electronic temperature ($\theta_{el}=0$) due to its low computational cost and reasonable accuracy [2,3,4,5]. Conventional time-dependent density functional theory (TD-DFT) [6] (also called the time-dependent Kohn–Sham (TDKS) method, real-time TD-DFT (RT-TD-DFT), or real-time density functional theory (RT-DFT)), which is the time-dependent (TD) extension of KS-DFT, has been recently applied to explore the TD and excited-state properties of electronic systems under the influence of TD external potentials [7,8,9]. Recently, a frequency-domain formulation of linear-response TD-DFT (LR-TD-DFT) [10] has also been adopted to obtain excitation energies (i.e., limited to the weak-field perturbative regime), owing to its computational efficiency and reasonable accuracy [7,8,9]. Nevertheless, for the study of TD phenomena or excitation energies beyond the linear response, conventional TD-DFT [6], which involves propagating the TDKS equation in the time domain without any restriction to the TD external potentials, remains a promising method.

In KS-DFT [1], since the exact exchange-correlation (xc) energy functional $E_{\mathrm{xc}}\left[\rho \right]$, in terms of the GS density ρ(r), has not been discovered, it remains necessary to adopt density functional approximations (DFAs) for $E_{\mathrm{xc}}\left[\rho \right]$ to perform practical calculations [2,3,4,5]. The xc energy functionals based on the frequently adopted DFAs, such as the LDA (local density approximation) [11,12] and GGAs (generalized gradient approximations) [13], are computationally efficient for the study of large systems. However, the DFA xc energy functionals have a few intrinsic shortcomings [2,3,4,5] and can yield the following qualitative errors: the self-interaction error (SIE), non-covalent interaction error (NCIE), and static correlation error (SCE). Since conventional TD-DFT [6], which usually takes the GS of a physical system as the initial state and often employs the GS xc potential (i.e., the functional derivative of $E_{\mathrm{xc}}\left[\rho \right]$) evaluated at the instantaneous density ρ(r,t) in the so-called adiabatic approximation [7,8,9], the qualitative errors of $E_{\mathrm{xc}}\left[\rho \right]$ can also degrade the accuracy of conventional TD-DFT results [14,15,16,17].

These qualitative errors can generally be reduced with the modification of the DFA functionals. For example, the SIE can be reduced by mixing the Hartree–Fock (HF) exchange energy into the parent DFA functionals (commonly called hybrid functionals) [18,19,20]. The NCIE can be reduced by combining the parent DFA functionals with the dispersion energy correction (also known as dispersion-corrected functionals) [21,22] or with the second-order Møller–Plesset (MP2) correlation energy (often called double-hybrid functionals) [23]. The SCE can be reduced by incorporating a fully nonlocal correlation energy component, such as the RPA (random phase approximation) correlation energy [24,25], into the parent DFA functionals. Nonetheless, the DFA, dispersion-corrected, hybrid, and double-hybrid functionals fail to resolve the SCE problem, while the RPA and related functionals are very demanding in computational expense and hence are impractical for large systems.

To circumvent the SCE problem at low computational cost, thermally assisted occupation density functional theory (TAO-DFT) [26] (i.e., a density functional theory with fractional orbital occupations) has been recently developed. Note that TAO-DFT is an electronic structure method for the GS properties of physical systems at zero electronic temperature ($\theta_{el}=0$), even though it adopts a reference system of noninteracting electrons at some fictitious temperature θ. The xc energy functionals developed in KS-DFT can also be used in TAO-DFT [26,27,28,29]. Nonetheless, in strong contrast to KS-DFT, TAO-DFT, even with the commonly used DFA, dispersion-corrected, and hybrid functionals, can approximately describe strong static correlation effects, especially when an appropriate value of θ is chosen [26,27,28]. Consequently, TAO-DFT is very promising for studying the GS properties of large systems with strong static correlation effects [30,31,32,33,34,35,36,37]. Other TAO-DFT extensions include the schemes that determine the system-independent [38] and system-dependent [39] values of θ, TAO-DFT-based ab initio molecular dynamics (for equilibrium thermodynamic and dynamical properties) [40], and TAO-DFT-based polarizable continuum model (for solvation effects) [41].

Within the framework of TAO-DFT, Yeh et al. have recently proposed a frequency-domain formulation of linear-response time-dependent TAO-DFT [42], denoted as TDTAO-DFT (or, more precisely, LR-TDTAO-DFT by its inherent linear-response (LR) nature), allowing excitation energy calculations in the frequency domain (i.e., using Casida’s formulation [10]). In TDTAO-DFT, the TD effective one-electron potential (see Equations (6) and (B6) of Ref. [42]) is defined with the TD pure state $|\Psi_{\mathrm{TAO}}\left(t\right)\rangle$ of a noninteracting reference system (also see Appendix B1 of Ref. [42]). However, the TD density ρ(r,t) (see Equation (5) of Ref. [42]) in TDTAO-DFT is generally not associated with a TD noninteracting pure state $|\Psi_{\mathrm{TAO}}\left(t\right)\rangle$ but associated with a TD noninteracting ensemble (which should be described by a TD density operator, as will be discussed later). For example, in TDTAO-DFT (with θ≠0), at the initial time $t_{0}$, the initial density $\rho (r,t_{0})$ is simply the TAO-DFT GS density ρ(r) (see Equation (1) of Ref. [42]), which should be associated with a thermal ensemble [26] (i.e., not associated with a pure state) of noninteracting electrons at a nonvanishing fictitious temperature (θ≠0). Therefore, the underlying assumption of TDTAO-DFT (i.e., that the TD density ρ(r,t) is assumed to be associated with the TD pure state $|\Psi_{\mathrm{TAO}}\left(t\right)\rangle$ of a noninteracting reference system) is generally incorrect, except only for the θ=0 case (wherein TDTAO-DFT reduces to conventional TD-DFT [6] or, more precisely, LR-TD-DFT [10] by its inherent LR nature).

To resolve the aforementioned inconsistency of TDTAO-DFT (especially for θ≠0) [42], in the work, we reformulate the TD extension of TAO-DFT by introducing a new reference system, consisting of an ensemble of noninteracting electrons moving in a TD local potential. This real-time (RT) extension of TAO-DFT is denoted as RT-TAO-DFT. Moreover, since the assumption of a weak perturbation is not required in RT-TAO-DFT, we also employ RT-TAO-DFT to study strong-field electron dynamics in molecules as well as high-order harmonic generation (HHG) [43,44,45,46,47,48,49,50,51,52,53,54,55,56,57,58,59,60].

The rest of this paper is organized as follows. In Section 2, we review TAO-DFT and discuss closely related electronic structure methods. The formulation of RT-TAO-DFT is presented in Section 3. In Section 4, we describe the details of our RT-TAO-DFT calculations for the HHG spectra and related TD properties of molecular hydrogen $H_{2}$ at the equilibrium and stretched geometries, aligned along the polarization of an intense linearly polarized laser pulse. The TD properties computed using RT-TAO-DFT are discussed and compared with the results of conventional TD-DFT [6]. Moreover, issues related to the possible spin-symmetry breaking effects in the TD properties are also discussed. Our conclusions are provided in Section 5.

## 2. Ground-State Theory: TAO-DFT

### 2.1. Overview of TAO-DFT

Consider a physical system of *N* interacting electrons moving in an external potential $v_{\mathrm{ext}}\left(r\right)$ at zero electronic temperature ($\theta_{el}=0$). In TAO-DFT [26], the GS density ρ(r) of the physical system is represented by the thermal equilibrium density of a reference system (called the thermally assisted occupation (TAO) reference system) of noninteracting electrons in the presence of a local potential $v_{\mathrm{TAO}}\left(r\right)$ (called the TAO potential) at some fictitious temperature θ (i.e., the temperature of the TAO reference system). In other words, ρ(r) is represented by the TAO orbitals $\left\{\phi_{j}\left(r\right)\right\}$ and TAO orbital occupation numbers (TOONs) $\left\{f_{j}\right\}$ (atomic units (a.u.) are adopted throughout this work):

(1) $$\rho \left(r\right)=\sum_{j}f_{j}{\left|\phi_{j}\left(r\right)\right|}^{2}.$$

Here, $f_{j}$ is the occupation number of the *j*-th TAO orbital $\phi_{j}\left(r\right)$, given by the Fermi–Dirac (FD) distribution function

(2) $$f_{j}={\left\{1+\exp\left[\left(\epsilon_{j}-\mu \right)/\theta \right]\right\}}^{-1},$$

where $0\leq f_{j}\leq 1$, $\epsilon_{j}$ is the energy of the *j*-th TAO orbital $\phi_{j}\left(r\right)$ and μ is the chemical potential chosen to conserve *N* (i.e., the number of electrons):

(3) $$\sum_{j}{\left\{1+\exp\left[\left(\epsilon_{j}-\mu \right)/\theta \right]\right\}}^{-1}=N.$$

On the basis of the Hohenberg–Kohn (HK) theorems [61] for the physical system at $\theta_{el}=0$ and the Mermin theorems [62] for the TAO reference system at the fictitious temperature θ, a set of self-consistent equations (i.e., the TAO equations) that determine the TAO orbitals $\left\{\phi_{j}\left(r\right)\right\}$, the TAO orbital energies $\left\{\epsilon_{j}\right\}$, and hence the TOONs $\left\{f_{j}\right\}$ (see Equations (2) and (3)) and the GS density ρ(r) (see Equation (1)) are given by [26]

(4) $${\hat{h}}_{\mathrm{TAO}}\left(r\right)\phi_{j}\left(r\right)=\epsilon_{j}\phi_{j}\left(r\right).$$

Here, ${\hat{h}}_{\mathrm{TAO}}\left(r\right)$ is the TAO effective one-electron Hamiltonian:

(5) $${\hat{h}}_{\mathrm{TAO}}\left(r\right)=-\frac{1}{2}\nabla_{r}^{2}\;+\;v_{\mathrm{TAO}}\left(r\right),$$

with the TAO potential (i.e., the TAO effective one-electron potential)

(6) $$\begin{array}{cc} v_{\mathrm{TAO}}\left(r\right)= & \;v_{\mathrm{ext}}\left(r\right)+\frac{\delta E_{H}\left[\rho \right]}{\delta \rho (r)}+\frac{\delta E_{\mathrm{xc}\theta }\left[\rho \right]}{\delta \rho (r)}\\ = & \;v_{\mathrm{ext}}\left(r\right)+v_{H}\left(r\right)+v_{\mathrm{xc}\theta }\left(r\right), \end{array}$$

where $v_{\mathrm{ext}}\left(r\right)$ is the external potential of the physical system, $v_{H}\left(r\right)=\frac{\delta E_{H}\left[\rho \right]}{\delta \rho (r)}=\int dr^{\prime }\frac{\rho (r^{\prime })}{|r-r^{\prime }|}$ is the Hartree potential (i.e., the functional derivative of the Hartree energy functional $E_{H}\left[\rho \right]=\frac{1}{2}\int dr\int dr^{\prime }\frac{\rho \left(r\right)\rho \left(r^{\prime }\right)}{|r-r^{\prime }|}$), and $v_{\mathrm{xc}\theta }\left(r\right)=\frac{\delta E_{\mathrm{xc}\theta }\left[\rho \right]}{\delta \rho (r)}$ is the xc*θ* potential, which is the functional derivative of the xc*θ* energy functional $E_{\mathrm{xc}\theta }\left[\rho \right]=E_{\mathrm{xc}}\left[\rho \right]+E_{\theta }\left[\rho \right]$, with $E_{\mathrm{xc}}\left[\rho \right]$ being the xc energy functional (as defined in KS-DFT [1]) and $E_{\theta }\left[\rho \right]$ being the θ-dependent energy functional (e.g., see Equation (14) of Ref. [26]).

In TAO-DFT [26], to obtain the GS density ρ(r) (i.e., represented by Equation (1) with the TAO orbitals $\left\{\phi_{j}\left(r\right)\right\}$ and TOONs $\left\{f_{j}\right\}$) of the physical system, Equation (1) to (6) should be solved self-consistently. After the self-consistency is achieved, the GS energy E[ρ] of the physical system (at $\theta_{el}=0$) is given by

(7) $$E\left[\rho \right]=\int dr\;\rho \left(r\right)v_{\mathrm{ext}}\left(r\right)+A_{s}^{\theta }\left[\left\{f_{j},\phi_{j}\right\}\right]+E_{H}\left[\rho \right]+E_{\mathrm{xc}\theta }\left[\rho \right],$$

where the first term is the external potential energy, $E_{H}\left[\rho \right]$ and $E_{\mathrm{xc}\theta }\left[\rho \right]$ are the Hartree and xc*θ* energy functionals, respectively, and $A_{s}^{\theta }\left[\left\{f_{j},\phi_{j}\right\}\right]$ is the noninteracting kinetic free energy at the fictitious temperature θ:

(8) $$A_{s}^{\theta }\left[\left\{f_{j},\phi_{j}\right\}\right]=T_{s}^{\theta }\left[\left\{f_{j},\phi_{j}\right\}\right]+E_{S}^{\theta }\left[\left\{f_{j}\right\}\right],$$

i.e., the sum of the kinetic energy

(9) $$T_{s}^{\theta }\left[\left\{f_{j},\phi_{j}\right\}\right]=-\frac{1}{2}\sum_{j}f_{j}\int dr\;\phi_{j}^{*}\left(r\right)\nabla_{r}^{2}\phi_{j}\left(r\right)$$

and entropy contribution

(10) $$E_{S}^{\theta }\left[\left\{f_{j}\right\}\right]=\theta \sum_{j}\left[f_{j}\;\ln\left(f_{j}\right)+\left(1-f_{j}\right)\;\ln\left(1-f_{j}\right)\right]$$

of noninteracting electrons at the fictitious temperature θ, which can be exactly computed using the TAO orbitals $\left\{\phi_{j}\left(r\right)\right\}$ and TOONs $\left\{f_{j}\right\}$. Note that, for the special case of θ=0, $E_{\theta =0}\left[\rho \right]=0$ and TAO-DFT (with $E_{\mathrm{xc}\theta }\left[\rho \right]$) [26] reduce to KS-DFT (with $E_{\mathrm{xc}}\left[\rho \right]$) [1].

### 2.2. Density Representation in TAO-DFT

The GS density ρ(r) of a physical system is interacting *v*-representable (I-VR) as the exact ρ(r) belongs to a GS wavefunction of an interacting *N*-electron Hamiltonian for some external potential $v_{\mathrm{ext}}\left(r\right)$, which can be exactly computed using the full configuration interaction (FCI) method at the complete basis set limit [63]. Moreover, the exact ρ(r) can be represented by the natural orbitals $\left\{\chi_{j}\left(r\right)\right\}$ and natural orbital occupation numbers (NOONs) $\left\{n_{j}\right\}$ [64]:

(11) $$\rho_{\mathrm{FCI}}\left(r\right)=\sum_{j}n_{j}{\left|\chi_{j}\left(r\right)\right|}^{2},$$

where the NOONs $\left\{n_{j}\right\}$ satisfy the following conditions,

(12) $$0\leq n_{j}\leq 1,\;\;\sum_{j}n_{j}=N.$$

Nevertheless, in KS-DFT [1], ρ(r) is assumed to be noninteracting pure-state *v*-representable (NI-PS-VR) as it belongs to a one-determinantal GS wavefunction of a noninteracting *N*-electron Hamiltonian (i.e., the Kohn–Sham (KS) Hamiltonian) for some local potential (i.e., the KS potential) [65,66,67]. Accordingly, in KS-DFT, ρ(r) is represented by the occupied KS orbitals $\left\{\phi_{i}^{\mathrm{KS}}\left(r\right)\right\}$:

(13) $$\rho_{\mathrm{KS}}\left(r\right)=\sum_{i=1}^{N}{\left|\phi_{i}^{\mathrm{KS}}\left(r\right)\right|}^{2}.$$

As has been shown in a number of studies [66,67,68,69,70,71], there are some reasonable GS densities (e.g., the GS densities of some electronic systems with strong static correlation effects) that are not NI-PS-VR. Apparently, these GS densities cannot be obtained with KS-DFT even adopting the exact xc energy functional $E_{\mathrm{xc}}\left[\rho \right]$.

On the other hand, in TAO-DFT [26], ρ(r) (given by Equation (1)) is assumed to be noninteracting thermal ensemble *v*-representable (NI-TE-VR) as it belongs to a thermal ensemble of a reference system of noninteracting electrons in the presence of a local potential (i.e., the TAO potential) at some fictitious temperature θ. Consequently, in TAO-DFT, ρ(r) is represented by the TAO orbitals $\left\{\phi_{j}\left(r\right)\right\}$ and TOONs $\left\{f_{j}\right\}$:

(14) $$\rho_{\mathrm{TAO}}\left(r\right)=\sum_{j}f_{j}{\left|\phi_{j}\left(r\right)\right|}^{2}.$$

where the TOONs $\left\{f_{j}\right\}$ (given by the FD distribution function) satisfy the following conditions,

(15) $$0\leq f_{j}\leq 1,\;\;\sum_{j}f_{j}=N.$$

Owing to the similar expressions of the TAO-DFT GS density $\rho_{\mathrm{TAO}}\left(r\right)$ (see Equation (14)) and the exact GS density $\rho_{\mathrm{FCI}}\left(r\right)$ (see Equation (11)), the fictitious temperature θ in TAO-DFT can be so chosen that the distribution of TOONs is close to the distribution of the exact NOONs, which is closely related to the stability (i.e., the single-reference (SR)/multi-reference (MR) character) of the GS of an electronic system [26]. Accordingly, the exact GS density is more likely to be NI-TE-VR with this choice of θ. In contrast to KS-DFT (i.e., TAO-DFT with θ=0), TAO-DFT has an extra degree of freedom in choosing the θ value to improve the GS density representability.

### 2.3. Approximate Energy Functionals and Fictitious Temperatures in TAO-DFT

Since the exact xc*θ* energy functional $E_{\mathrm{xc}\theta }\left[\rho \right]$ (i.e., one of the key ingredients in TAO-DFT), in terms of the GS density ρ(r), has not been known, it remains necessary to employ DFAs for $E_{\mathrm{xc}\theta }\left[\rho \right]$ to perform practical calculations using TAO-DFT. Conventional DFAs, such as the LDA and GGAs, for $E_{\mathrm{xc}\theta }\left[\rho \right]$ (i.e., the DFA xc*θ* energy functional $E_{\mathrm{xc}\theta }^{\mathrm{DFA}}\left[\rho \right]$) can be adopted [26,27]. In addition to TAO-DFA (i.e., TAO-DFT with the DFA functional $E_{\mathrm{xc}\theta }^{\mathrm{DFA}}\left[\rho \right]$), TAO-DFT with the exact exchange [28] and related hybrid functionals [28,29] may also be employed.

For the GS of an electronic system, the fictitious temperature θ of a given energy functional in TAO-DFT should be so selected that the distribution of TOONs simulates the distribution of the exact NOONs. In this situation, the static correlation associated with the electronic GS can be properly captured by the entropy contribution (see Equation (10)) in TAO-DFT [26]. In other words, the optimal θ should be closely related to the SR/MR character of the electronic GS. For systems with electronic ground states possessing SR character (i.e., SR systems), all the NOONs should be close to either 0 (fully empty) or 1 (fully occupied), and, hence, the optimal θ values in TAO-DFT should be sufficiently small (but nonvanishing for real electronic systems [38]). However, for systems with electronic ground states possessing MR character (i.e., MR systems), the distributions of NOONs (and hence the optimal θ values) can be highly system-dependent. While it remains very challenging to devise a scheme that always yields the best θ of each system for a given energy functional in TAO-DFT, some progress has been achieved in recent years.

For a given energy functional in TAO-DFT, if the optimal θ values of electronic systems can be kept within a narrow range of values, it would be very useful to define an optimal system-independent θ value. Recently, TAO-DFT with the optimal system-independent θ scheme [26,27,28,38], which is as efficient as KS-DFT (i.e., TAO-DFT with θ=0) in computational cost, can be comparable to KS-DFT in performance for various SR systems [26,27,28,38,41], and can outperform KS-DFT for several MR systems [26,27,28,30,31,32,33,34,35,36,37,38,40,41]. To improve the optimal system-independent θ scheme, a self-consistent scheme that determines the optimal θ values of electronic systems has been recently proposed [39].

### 2.4. Comparison of KS-DFT, TAO-DFT, and FT-DFT

Here, we compare three generally different electronic structure methods (see Table 1): KS-DFT [1], TAO-DFT [26], and FT-DFT (finite-temperature density functional theory, also called the Mermin–Kohn–Sham (MKS) method) [1,62], each of which employs a reference system of noninteracting electrons in the presence of a local potential at some fictitious temperature θ.

Both KS-DFT and TAO-DFT are electronic structure methods for the GS properties of physical systems at zero electronic temperature ($\theta_{el}=0$). Note that $\theta \equiv \theta_{el}=0$ is assumed in KS-DFT, while the fictitious temperature (θ≥0) can be different from the electronic temperature ($\theta_{el}=0$) in TAO-DFT. Accordingly, the GS density ρ(r) of a physical system at $\theta_{el}=0$ is assumed to be NI-PS-VR in KS-DFT and NI-TE-VR in TAO-DFT. Moreover, in KS-DFT, the HK universal functional (i.e., the sum of the interacting kinetic energy and the electron–electron repulsion energy at $\theta_{el}=0$) [61], which is a functional of the GS density ρ(r), is given by

(16) $$F_{\mathrm{HK}}\left[\rho \right]=T_{s}\left[\left\{\phi_{i}^{\mathrm{KS}}\right\}\right]+E_{H}\left[\rho \right]+E_{\mathrm{xc}}\left[\rho \right],$$

where $T_{s}\left[\left\{\phi_{i}^{\mathrm{KS}}\right\}\right]$ (exactly computed using the occupied KS orbitals $\left\{\phi_{i}^{\mathrm{KS}}\left(r\right)\right\}$) is the noninteracting kinetic energy at zero fictitious temperature (θ=0), and $E_{\mathrm{xc}}\left[\rho \right]$ is the xc energy functional, which needs to be approximated for practical KS-DFT calculations. By contrast, in TAO-DFT, the HK universal functional [61], which is a functional of the GS density ρ(r), is expressed as

(17) $$F_{\mathrm{HK}}\left[\rho \right]=A_{s}^{\theta }\left[\left\{f_{j},\phi_{j}\right\}\right]+E_{H}\left[\rho \right]+E_{\mathrm{xc}\theta }\left[\rho \right],$$

where $A_{s}^{\theta }\left[\left\{f_{j},\phi_{j}\right\}\right]$ (exactly computed using the TAO orbitals $\left\{\phi_{j}\left(r\right)\right\}$ and TOONs $\left\{f_{j}\right\}$) is the noninteracting kinetic free energy at the fictitious temperature θ, and $E_{\mathrm{xc}\theta }\left[\rho \right]$ is the xc*θ* energy functional, which needs to be approximated for practical TAO-DFT calculations. Note that TAO-DFT (with θ=0) reduces to KS-DFT.

On the other hand, FT-DFT (i.e., the MKS method) is an electronic structure method for the thermal equilibrium properties of physical systems at finite electronic temperatures ($\theta_{el}\geq 0$), and $\theta \equiv \theta_{el}$ is assumed in FT-DFT. Therefore, the thermal equilibrium density $\rho^{\theta_{el}}\left(r\right)$ of a physical system at $\theta_{el}$ is assumed to be NI-TE-VR in FT-DFT. Moreover, in FT-DFT, the Mermin (M) universal functional (i.e., the sum of the interacting kinetic free energy and the electron–electron repulsion energy at $\theta_{el}$) [62], which is a $\theta_{el}$-dependent functional of the thermal equilibrium density $\rho^{\theta_{el}}\left(r\right)$, is given by

(18) $$F_{M}^{\theta_{el}}\left[\rho^{\theta_{el}}\right]=A_{s}^{\theta_{el}}\left[\left\{f_{k}^{\mathrm{MKS}},\phi_{k}^{\mathrm{MKS}}\right\}\right]+E_{H}\left[\rho^{\theta_{el}}\right]+F_{\mathrm{xc}}^{\theta_{el}}\left[\rho^{\theta_{el}}\right],$$

where $A_{s}^{\theta_{el}}\left[\left\{f_{k}^{\mathrm{MKS}},\phi_{k}^{\mathrm{MKS}}\right\}\right]$ (exactly computed using the MKS orbitals $\left\{\phi_{k}^{\mathrm{MKS}}\left(r\right)\right\}$ and MKS orbital occupation numbers $\left\{f_{k}^{\mathrm{MKS}}\right\}$) are the noninteracting kinetic free energy at the fictitious temperature $\theta \equiv \theta_{el}$, and $F_{\mathrm{xc}}^{\theta_{el}}\left[\rho^{\theta_{el}}\right]$ is the xc free energy functional, which needs to be approximated for practical FT-DFT calculations. Note that FT-DFT (with $\theta_{el}=0$) reduces to KS-DFT.

Consequently, for the GS properties of physical systems at $\theta_{el}=0$, FT-DFT reduces to KS-DFT, while TAO-DFT (with θ≠0) can be very different from KS-DFT (especially for MR systems) [26,38,41].

### 2.5. TAO-DFT-Related Methods

#### 2.5.1. TAO-DFT with $E_{\mathrm{xc}\theta }\left[\rho \right]\approx E_{\mathrm{xc}}\left[\rho \right]$

Here, we compare two approximate methods (see Table 2) that are closely related to TAO-DFT [26] and FT-DFT (i.e., the MKS method) [1,62].

As mentioned previously, TAO-DFT is an electronic structure method for the GS properties of physical systems at zero electronic temperature ($\theta_{el}=0$). In TAO-DFT, the xc*θ* energy functional $E_{\mathrm{xc}\theta }\left[\rho \right]=E_{\mathrm{xc}}\left[\rho \right]+E_{\theta }\left[\rho \right]$. At zero fictitious temperature (θ=0), $E_{\theta =0}\left[\rho \right]=0$ and hence $E_{\mathrm{xc}\theta }\left[\rho \right]=E_{\mathrm{xc}}\left[\rho \right]$. At a sufficiently small fictitious temperature (θ≈0), the magnitude of $E_{\theta \approx 0}\left[\rho \right]$ should remain small compared to that of $E_{\mathrm{xc}}\left[\rho \right]$, and hence the approximation $E_{\mathrm{xc}\theta }\left[\rho \right]\approx E_{\mathrm{xc}}\left[\rho \right]$ can be reasonably justified. Clearly, TAO-DFT with $E_{\mathrm{xc}\theta }\left[\rho \right]\approx E_{\mathrm{xc}}\left[\rho \right]$ (also called TAO-DFT without $E_{\theta }\left[\rho \right]$) is an approximate TAO-DFT method (good for θ≈0), which may be adopted to describe the strong static correlation effects of some GS systems (wherever θ≈0 can be an appropriate fictitious temperature) at $\theta_{el}=0$.

On the other hand, FT-DFT is an electronic structure method for the thermal equilibrium properties of physical systems at finite electronic temperatures ($\theta_{el}\geq 0$), wherein $\theta \equiv \theta_{el}$ is assumed. In FT-DFT, at zero electronic temperature ($\theta_{el}=0$), $F_{\mathrm{xc}}^{\theta_{el}=0}\left[\rho^{\theta_{el}=0}\right]=E_{\mathrm{xc}}\left[\rho \right]$. At a sufficiently small electronic temperature ($\theta_{el}\approx 0$), the approximation $F_{\mathrm{xc}}^{\theta_{el}}\left[\rho^{\theta_{el}}\right]\approx E_{\mathrm{xc}}\left[\rho^{\theta_{el}}\right]$ can be reasonably justified. Apparently, FT-DFT with $F_{\mathrm{xc}}^{\theta_{el}}\left[\rho^{\theta_{el}}\right]\approx E_{\mathrm{xc}}\left[\rho^{\theta_{el}}\right]$ is an approximate FT-DFT method (good for $\theta \equiv \theta_{el}\approx 0$), which may be used to study the temperature effects of thermal equilibrium systems at $\theta_{el}\approx 0$ [72,73].

According to their mathematical expressions, TAO-DFT with $E_{\mathrm{xc}\theta }\left[\rho \right]\approx E_{\mathrm{xc}}\left[\rho \right]$ [26] is strikingly similar to FT-DFT with $F_{\mathrm{xc}}^{\theta_{el}}\left[\rho^{\theta_{el}}\right]\approx E_{\mathrm{xc}}\left[\rho^{\theta_{el}}\right]$ [1,62]. However, owing to their distinctly different physical meanings, one can easily distinguish the two approximate methods simply based on the electronic properties computed. For example, for the GS properties of physical systems at $\theta_{el}=0$, FT-DFT with $F_{\mathrm{xc}}^{\theta_{el}}\left[\rho^{\theta_{el}}\right]\approx E_{\mathrm{xc}}\left[\rho^{\theta_{el}}\right]$ reduces to KS-DFT with $E_{\mathrm{xc}}\left[\rho \right]$, while TAO-DFT with $E_{\mathrm{xc}\theta }\left[\rho \right]\approx E_{\mathrm{xc}}\left[\rho \right]$ at a nonvanishing fictitious temperature (θ≠0) can be very different from KS-DFT with $E_{\mathrm{xc}}\left[\rho \right]$ (especially for MR systems) [26,38,41]. Therefore, a number of recent studies on the GS properties of physical systems at $\theta_{el}=0$ [74,75,76,77,78,79,80,81] have actually been performed using TAO-DFT with $E_{\mathrm{xc}\theta }\left[\rho \right]\approx E_{\mathrm{xc}}\left[\rho \right]$ [26] rather than FT-DFT with $F_{\mathrm{xc}}^{\theta_{el}}\left[\rho^{\theta_{el}}\right]\approx E_{\mathrm{xc}}\left[\rho^{\theta_{el}}\right]$ (which, in fact, should reduce to KS-DFT with $E_{\mathrm{xc}}\left[\rho \right]$ at $\theta_{el}=0$) [1,62].

#### 2.5.2. KS-DFA with the rTAO Energy Correction

On the basis of the TAO-DFA (with some fictitious temperature θ) energy expression [26,27], Yeh, Yang, and Hsu have recently proposed a post-KS energy correction, called the rTAO energy correction [82], which is a θ-dependent energy correction evaluated with the KS-DFA (i.e., KS-DFT with the DFA xc energy functional) orbitals. Owing to the post-KS nature, for clarity, we denote this method as the KS-DFA+rTAO method.

For a small fictitious temperature (θ≲40 mhartree), the KS-DFA+rTAO method has been shown to approximately reproduce the TAO-DFA results for some selected electronic properties [82]. This may in part be due to the post-KS nature (i.e., good for small θ) and a very limited amount of test data. For a considerably large θ (e.g., the optimal θ of TAO-DFT with the exact exchange for the dissociation of molecular hydrogen $H_{2}$ [28,38]) or for other electronic properties (e.g., atomization energies), the results obtained with this method can be very different from those obtained with TAO-DFT.

More importantly, the GS density obtained with the KS-DFA+rTAO method (with any value of θ) is the same as the KS-DFA GS density (i.e., NI-PS-VR). In other words, some reasonable non-NI-PS-VR GS densities (e.g., the GS densities of some MR systems) cannot be obtained with KS-DFA and the KS-DFA+rTAO method (with any value of θ) [66,67,68,69,70,71].

In particular, whenever the spin-symmetry constraint [3,4,26,83] on the singlet GS density of an electronic system is violated with KS-DFA (which can commonly happen for MR systems), it must also be violated with the KS-DFA+rTAO method (with any value of θ). In such a situation, the spin-unrestricted KS-DFA/KS-DFA+rTAO results can be very different from the corresponding spin-restricted KS-DFA/KS-DFA+rTAO results, yielding unphysical spin-symmetry breaking effects in the spin-unrestricted KS-DFA/KS-DFA+rTAO calculations. By contrast, the spin-symmetry breaking issues can be greatly resolved by TAO-DFA (with an appropriate θ) [26,27,28,30,33,38,39,40,41], highlighting the significance of the GS density representation in TAO-DFT.

## 3. Real-Time Theory: RT-TAO-DFT

### 3.1. RT-TAO Equation

Consider a physical system of *N* interacting electrons moving in a TD external potential $v_{\mathrm{ext}}\left(r,t\right)$. The Hamiltonian operator of the physical system is given by

(19) $$\hat{H}\left(t\right)=\hat{T}+{\hat{V}}_{\mathrm{ee}}+{\hat{v}}_{\mathrm{ext}}\left(t\right),$$

containing the operators of the kinetic energy

(20) $$\hat{T}=-\frac{1}{2}\sum_{i=1}^{N}\nabla_{r_{i}}^{2},$$

the electron–electron interaction

(21) $${\hat{V}}_{\mathrm{ee}}=\sum_{i=1}^{N}\sum_{j>i}^{N}\frac{1}{|r_{i}-r_{j}|},$$

and the TD external potential

(22) $${\hat{v}}_{\mathrm{ext}}\left(t\right)=\sum_{i=1}^{N}v_{\mathrm{ext}}\left(r_{i},t\right).$$

Let |Ψ(t)〉 be the TD state of the physical system. For most TD cases of physical interest, in this work, the time propagation is assumed to start from the GS $|\Psi_{\mathrm{GS}}\rangle$ (i.e., the lowest energy eigenstate of $\hat{H}\left(t_{0}\right)$, which is a stationary state) of the unperturbed physical system at time $t=t_{0}$, and $|\Psi_{\mathrm{GS}}\rangle$ is assumed to be non-degenerate. Accordingly, the TD state |Ψ(t)〉 of the physical system is a solution of the TD Schrödinger equation (TDSE):

(23) $$i\frac{\partial }{\partial t}|\Psi \left(t\right)\rangle =\hat{H}\left(t\right)|\Psi \left(t\right)\rangle ,$$

with the initial state

(24) $$|\Psi \left(t_{0}\right)\rangle =|\Psi_{\mathrm{GS}}\rangle .$$

From the TD state |Ψ(t)〉, the TD density ρ(r,t) of the physical system can be determined by

(25) $$\rho \left(r,t\right)=\langle \Psi \left(t\right)|\hat{\rho }\left(r\right)|\Psi \left(t\right)\rangle ,$$

where $\hat{\rho }\left(r\right)=\sum_{i=1}^{N}\delta \left(r-r_{i}\right)$ is the number density operator. In particular, the initial density $\rho (r,t_{0})$ of the physical system is given by the GS density $\rho_{\mathrm{GS}}\left(r\right)$ of the unperturbed physical system:

(26) $$\rho \left(r,t_{0}\right)=\rho_{\mathrm{GS}}\left(r\right)=\langle \Psi_{\mathrm{GS}}|\hat{\rho }\left(r\right)|\Psi_{\mathrm{GS}}\rangle .$$

According to the Runge–Gross (RG) theorems for the physical system (i.e., consisting of a TD pure state) [6], the TD state |Ψ(t)〉 is a functional of the TD density ρ(r,t) (i.e., formally depending on the density $\rho (r,t^{\prime })$ at all previous times $t^{\prime }\leq t$) and the initial state $|\Psi (t_{0})\rangle$, i.e., $|\Psi \left(t\right)\rangle =|\Psi \left[\rho ,|\Psi \left(t_{0}\right)\rangle \right]\left(t\right)\rangle$. In this work, the initial state $|\Psi \left(t_{0}\right)\rangle =|\Psi_{\mathrm{GS}}\rangle$ is a functional of the initial density $\rho \left(r,t_{0}\right)=\rho_{\mathrm{GS}}\left(r\right)$ based on the HK theorems [61]. Since the dependence of initial state $|\Psi (t_{0})\rangle$ is implicitly included in the TD density ρ(r,t), for brevity, $|\Psi \left[\rho ,|\Psi \left(t_{0}\right)\rangle \right]\left(t\right)\rangle$ is denoted as |Ψ[ρ](t)〉 hereafter. Now, we define the action functional of the physical system:

(27) $$\begin{array}{cc} A[\rho ]= & \;\int_{t_{0}}^{t_{1}}dt\;\langle \Psi \left[\rho \right]\left(t\right)|\left(i\frac{\partial }{\partial t}-\hat{H}\left(t\right)\right)|\Psi \left[\rho \right]\left(t\right)\rangle \\ = & \;\int_{t_{0}}^{t_{1}}dt\;\langle \Psi \left[\rho \right]\left(t\right)|\left(i\frac{\partial }{\partial t}-\hat{T}-{\hat{V}}_{\mathrm{ee}}-{\hat{v}}_{\mathrm{ext}}\left(t\right)\right)|\Psi \left[\rho \right]\left(t\right)\rangle \\ = & \;B\left[\rho \right]-\int_{t_{0}}^{t_{1}}dt\int dr\;\rho \left(r,t\right)v_{\mathrm{ext}}\left(r,t\right), \end{array}$$

where B[ρ] is a universal functional of the TD density ρ(r,t):

(28) $$B\left[\rho \right]=\int_{t_{0}}^{t_{1}}dt\;\langle \Psi \left[\rho \right]\left(t\right)|\left(i\frac{\partial }{\partial t}-\hat{T}-{\hat{V}}_{\mathrm{ee}}\right)|\Psi \left[\rho \right]\left(t\right)\rangle .$$

Note that the action functional A[ρ] has a stationary point at the exact TD density ρ(r,t) of the physical system, given by the Euler equation:

(29) $$\frac{\delta A[\rho ]}{\delta \rho (r,t)}=\frac{\delta B[\rho ]}{\delta \rho (r,t)}-v_{\mathrm{ext}}\left(r,t\right)=0.$$

In order to develop an RT method compatible with TAO-DFT [26], we introduce the RT-TAO reference system, consisting of an ensemble of noninteracting electrons moving in a TD local potential $v_{s}\left(r,t\right)$. The RT-TAO reference system can interchange electrons with its environment, and, hence, the electron number $N_{e}$ in the RT-TAO reference system can be varied from 0 to *∞*. The Hamiltonian operator of the RT-TAO reference system is given by

(30) $${\hat{H}}_{s}\left(t\right)={\hat{T}}_{s}+{\hat{v}}_{s}\left(t\right),$$

containing the operators of the kinetic energy ${\hat{T}}_{s}$ and the TD local potential ${\hat{v}}_{s}\left(t\right)$.

At time $t=t_{0}$, the time propagation starts from the grand canonical ensemble (i.e., a stationary ensemble) of the TAO reference system at some fictitious temperature θ, obtained with TAO-DFT [26]. Therefore, the initial density operator ${\hat{\Gamma }}_{s}\left(t_{0}\right)$ of the RT-TAO reference system is given by the TAO density operator ${\hat{\Gamma }}_{\mathrm{TAO}}$ (i.e., the grand canonical density operator [84] of the TAO reference system at the fictitious temperature θ):

(31) $${\hat{\Gamma }}_{s}\left(t_{0}\right)={\hat{\Gamma }}_{\mathrm{TAO}}=\sum_{N_{e}}\sum_{n}w_{N_{e},n}|\Phi_{N_{e},n}^{0}\rangle \langle \Phi_{N_{e},n}^{0}|$$

with the equilibrium statistical weights

(32) $$w_{N_{e},n}=\frac{\exp[-\left(E_{N_{e},n}-\mu N_{e}\right)/\theta ]}{\sum_{N_{e}}\sum_{n}\exp\left[-\left(E_{N_{e},n}-\mu N_{e}\right)/\theta \right]},$$

satisfying the following conditions,

(33) $$0\leq w_{N_{e},n}\leq 1,\;\;\sum_{N_{e}}\sum_{n}w_{N_{e},n}=1.$$

Here, $|\Phi_{N_{e},n}^{0}\rangle$ denotes the *n*-th $N_{e}$-electron eigenstate of ${\hat{H}}_{s}\left(t_{0}\right)={\hat{H}}_{\mathrm{TAO}}$ (i.e., the Hamiltonian operator of the TAO reference system) and $E_{N_{e},n}$ the corresponding energy eigenvalue.

For $t>t_{0}$, the noninteracting ensemble of the RT-TAO reference system is generally non-stationary due to the presence of the TD local potential $v_{s}\left(r,t\right)$. According to the TDSE

(34) $$i\frac{\partial }{\partial t}|\Phi_{N_{e},n}\left(t\right)\rangle ={\hat{H}}_{s}\left(t\right)|\Phi_{N_{e},n}\left(t\right)\rangle ,$$

with the initial state $|\Phi_{N_{e},n}\left(t_{0}\right)\rangle =|\Phi_{N_{e},n}^{0}\rangle$, we know how the TD state $|\Phi_{N_{e},n}\left(t\right)\rangle$ evolves in time. Therefore, the TD noninteracting ensemble of the RT-TAO reference system can be properly defined by the following TD density operator ${\hat{\Gamma }}_{s}\left(t\right)$:

(35) $${\hat{\Gamma }}_{s}\left(t\right)=\sum_{N_{e}}\sum_{n}w_{N_{e},n}|\Phi_{N_{e},n},\left(t\right)\rangle \langle \Phi_{N_{e},n},\left(t\right)|,$$

where the statistical weights $w_{N_{e},n}$, which are assumed to be time-independent, are given by Equation (32), i.e., the initial statistical weights. Note that the TD density operator ${\hat{\Gamma }}_{s}\left(t\right)$ of the RT-TAO reference system is a solution of the Liouville–von Neumann equation:

(36) $$i\frac{\partial }{\partial t}{\hat{\Gamma }}_{s}\left(t\right)=\left[{\hat{H}}_{s}\left(t\right),{\hat{\Gamma }}_{s}\left(t\right)\right],$$

with the initial density operator ${\hat{\Gamma }}_{s}\left(t_{0}\right)={\hat{\Gamma }}_{\mathrm{TAO}}$ (given by Equation (31)). From the TD density operator ${\hat{\Gamma }}_{s}\left(t\right)$, the TD density $\rho_{s}\left(r,t\right)$ of the RT-TAO reference system can be determined by

(37) $$\rho_{s}\left(r,t\right)=\mathrm{Tr}\{{\hat{\Gamma }}_{s}\left(t\right){\hat{\rho }}_{s}\left(r\right)\}=\sum_{N_{e}}\sum_{n}w_{N_{e},n}\langle \Phi_{N_{e},n}\left(t\right)|{\hat{\rho }}_{s}\left(r\right)|\Phi_{N_{e},n}\left(t\right)\rangle ,$$

where ${\hat{\rho }}_{s}\left(r\right)$ is the number density operator [84]. In particular, the initial density $\rho_{s}\left(r,t_{0}\right)$ of the RT-TAO reference system is given by the thermal equilibrium density $\rho_{\mathrm{TAO}}\left(r\right)$ (see Equation (14)) of the TAO reference system [26], which is the same as the GS density $\rho_{\mathrm{GS}}\left(r\right)$ of the unperturbed physical system (note that $\rho_{\mathrm{GS}}\left(r\right)$ is assumed to be NI-TE-VR with this θ) and hence the initial density $\rho (r,t_{0})$ of the physical system (see Equation (26)):

(38) $$\begin{array}{cc} \rho_{s}\left(r,t_{0}\right)= & \;\rho_{\mathrm{TAO}}\left(r\right)=\mathrm{Tr}\left\{{\hat{\Gamma }}_{\mathrm{TAO}}{\hat{\rho }}_{s}\left(r\right)\right\}=\sum_{N_{e}}\sum_{n}w_{N_{e},n}\langle \Phi_{N_{e},n}^{0}|{\hat{\rho }}_{s}\left(r\right)|\Phi_{N_{e},n}^{0}\rangle \\ = & \;\rho_{\mathrm{GS}}\left(r\right)=\rho \left(r,t_{0}\right). \end{array}$$

Here, we seek $v_{s}\left(r,t\right)$ that yields the same solution $\rho_{s}\left(r,t\right)=\rho \left(r,t\right)$ for $t\geq t_{0}$. According to the Li–Tong (LT) theorems for the RT-TAO reference system (i.e., consisting of a TD noninteracting ensemble) [85], the TD density operator ${\hat{\Gamma }}_{s}\left(t\right)$ is a functional of the TD density $\rho_{s}\left(r,t\right)$ (i.e., formally depending on the density $\rho_{s}\left(r,t^{\prime }\right)$ at all previous times $t^{\prime }\leq t$) and the initial density operator ${\hat{\Gamma }}_{s}\left(t_{0}\right)$, i.e., ${\hat{\Gamma }}_{s}\left(t\right)={\hat{\Gamma }}_{s}\left[\rho_{s},{\hat{\Gamma }}_{s}\left(t_{0}\right)\right]\left(t\right)$. In this work, the initial density operator ${\hat{\Gamma }}_{s}\left(t_{0}\right)={\hat{\Gamma }}_{\mathrm{TAO}}$ (see Equation (31)) is a functional of the initial density $\rho_{s}\left(r,t_{0}\right)=\rho_{\mathrm{TAO}}\left(r\right)$ (see Equation (14)) based on the Mermin theorems [62]. Since the dependence of initial density operator ${\hat{\Gamma }}_{s}\left(t_{0}\right)$ is implicitly included in the TD density $\rho_{s}\left(r,t\right)$, for brevity, ${\hat{\Gamma }}_{s}\left[\rho_{s},{\hat{\Gamma }}_{s}\left(t_{0}\right)\right]\left(t\right)$ is denoted as ${\hat{\Gamma }}_{s}\left[\rho_{s}\right]\left(t\right)$ hereafter. Now, we define the action functional of the RT-TAO reference system:

(39) $$\begin{array}{cc} A_{s}\left[\rho_{s}\right]= & \;\int_{t_{0}}^{t_{1}}dt\;\mathrm{Tr}\left\{{\hat{\Gamma }}_{s}\left[\rho_{s}\right]\left(t\right)\left(i\frac{\partial }{\partial t}-{\hat{H}}_{s}\left(t\right)\right)\right\}\\ = & \;\int_{t_{0}}^{t_{1}}dt\;\mathrm{Tr}\left\{{\hat{\Gamma }}_{s}\left[\rho_{s}\right]\left(t\right)\left(i\frac{\partial }{\partial t}-{\hat{T}}_{s}-{\hat{v}}_{s}\left(t\right)\right)\right\}\\ = & \;B_{s}\left[\rho_{s}\right]-\int_{t_{0}}^{t_{1}}dt\int dr\;\rho_{s}\left(r,t\right)v_{s}\left(r,t\right), \end{array}$$

where $B_{s}\left[\rho_{s}\right]$ is a universal functional of the TD density $\rho_{s}\left(r,t\right)$:

(40) $$B_{s}\left[\rho_{s}\right]=\int_{t_{0}}^{t_{1}}dt\;\mathrm{Tr}\left\{{\hat{\Gamma }}_{s}\left[\rho_{s}\right]\left(t\right)\left(i\frac{\partial }{\partial t}-{\hat{T}}_{s}\right)\right\}$$

Note that the action functional $A_{s}\left[\rho_{s}\right]$ has a stationary point at the exact TD density $\rho_{s}\left(r,t\right)$ of the RT-TAO reference system, given by the Euler equation:

(41) $$\frac{\delta A_{s}\left[\rho_{s}\right]}{\delta \rho_{s}\left(r,t\right)}=\frac{\delta B_{s}\left[\rho_{s}\right]}{\delta \rho_{s}\left(r,t\right)}-v_{s}\left(r,t\right)=0.$$

In RT-TAO-DFT, the universal functional B[ρ] (given by Equation (28)) is partitioned as

(42) $$B\left[\rho \right]=B_{s}\left[\rho \right]-A_{H}\left[\rho \right]-A_{\mathrm{xc}\theta }\left[\rho \right],$$

where the universal functional $B_{s}\left[\rho \right]$ is given by Equation (40), $A_{H}\left[\rho \right]$ is the Hartree action functional:

(43) $$A_{H}\left[\rho \right]=\frac{1}{2}\int_{t_{0}}^{t_{1}}dt\int dr\int dr^{\prime }\;\frac{\rho \left(r,t\right)\rho \left(r^{\prime },t\right)}{|r-r^{\prime }|},$$

and $A_{\mathrm{xc}\theta }\left[\rho \right]$ is the xc*θ* action functional:

(44) $$A_{\mathrm{xc}\theta }\left[\rho \right]\equiv B_{s}\left[\rho \right]-B\left[\rho \right]-A_{H}\left[\rho \right],$$

which is a universal functional of the TD density ρ(r,t). Applying Equation (42) to Equation (29), we obtain

(45) $$\frac{\delta B_{s}\left[\rho \right]}{\delta \rho (r,t)}-\frac{\delta A_{H}\left[\rho \right]}{\delta \rho (r,t)}-\frac{\delta A_{\mathrm{xc}\theta }\left[\rho \right]}{\delta \rho (r,t)}-v_{\mathrm{ext}}\left(r,t\right)=0.$$

Comparing Equation (41) with Equation (45) shows that the same solution $\rho_{s}\left(r,t\right)=\rho \left(r,t\right)$ can be obtained if we choose the TD effective one-electron potential $v_{s}\left(r,t\right)$ (up to a purely TD function C(t)) of the RT-TAO reference system as

(46) $$\begin{array}{cc} v_{s}\left(r,t\right)= & \;v_{\mathrm{ext}}\left(r,t\right)+\frac{\delta A_{H}\left[\rho \right]}{\delta \rho (r,t)}+\frac{\delta A_{\mathrm{xc}\theta }\left[\rho \right]}{\delta \rho (r,t)}\\ = & \;v_{\mathrm{ext}}\left(r,t\right)+v_{H}\left(r,t\right)+v_{\mathrm{xc}\theta }\left(r,t\right), \end{array}$$

where $v_{\mathrm{ext}}\left(r,t\right)$ is the TD external potential of the physical system, $v_{H}\left(r,t\right)=\frac{\delta A_{H}\left[\rho \right]}{\delta \rho (r,t)}=\int dr^{\prime }\frac{\rho (r^{\prime },t)}{|r-r^{\prime }|}$ is the TD Hartree potential, and $v_{\mathrm{xc}\theta }\left(r,t\right)=\frac{\delta A_{\mathrm{xc}\theta }\left[\rho \right]}{\delta \rho (r,t)}$ is the TD xc*θ* potential.

Since the RT-TAO reference system consists of a TD noninteracting ensemble, the Hamiltonian ${\hat{H}}_{s}\left(t\right)$ (see Equation (30)) is separable, with the RT-TAO effective one-electron Hamiltonian

(47) $${\hat{h}}_{s}\left(r,t\right)=-\frac{1}{2}\nabla_{r}^{2}+v_{s}\left(r,t\right),$$

where the TD effective one-electron potential $v_{s}\left(r,t\right)$ (denoted as the RT-TAO potential) is given by Equation (46). The RT-TAO orbitals $\left\{\phi_{j}\left(r,t\right)\right\}$ evolve in time according to the effective one-electron TDSE (denoted as the RT-TAO equation):

(48) $$i\frac{\partial }{\partial t}\phi_{j}\left(r,t\right)={\hat{h}}_{s}\left(r,t\right)\phi_{j}\left(r,t\right),$$

with

(49) $$\phi_{j}\left(r,t_{0}\right)=\phi_{j}^{0}\left(r\right),$$

i.e., the initial *j*-th RT-TAO orbital $\phi_{j}\left(r,t_{0}\right)$ is given by the *j*-th TAO orbital $\phi_{j}^{0}\left(r\right)$ (the *j*-th energy eigenfunction of ${\hat{h}}_{s}\left(r,t_{0}\right)={\hat{h}}_{\mathrm{TAO}}\left(r\right)$ (see Equation (5)), associated with the GS of the unperturbed physical system) [26]. The TD density ρ(r,t) of the physical system, which is the same as the TD density $\rho_{s}\left(r,t\right)$ (given by Equation (37)) of the RT-TAO reference system, can be computed using [84]

(50) $$\rho \left(r,t\right)=\rho_{s}\left(r,t\right)=\sum_{j}f_{j}{\left|\phi_{j}\left(r,t\right)\right|}^{2},$$

where the occupation number $f_{j}$ of the *j*-th RT-TAO orbital $\phi_{j}\left(r,t\right)$, which is time-independent, is given by Equation (2), i.e., its initial occupation number [26], also satisfying the conditions $0\leq f_{j}\leq 1$ and $\sum_{j}f_{j}=N$.

For the special case of θ=0, RT-TAO-DFT (with the xc*θ* action functional $A_{\mathrm{xc}\theta }\left[\rho \right]$) reduces to conventional TD-DFT (with the xc action functional $A_{\mathrm{xc}}\left[\rho \right]$) [6], providing that, at time $t=t_{0}$, the initial state of the physical system is the non-degenerate GS of the unperturbed physical system.

Here, we discuss the representation of the TD density ρ(r,t) of a physical system in conventional TD-DFT [6] and RT-TAO-DFT. In conventional TD-DFT, ρ(r,t) is assumed to be TD noninteracting pure-state *v*-representable (TD-NI-PS-VR) as it belongs to a TD one-determinantal wavefunction of a noninteracting *N*-electron Hamiltonian for some TD local potential. By contrast, in RT-TAO-DFT, ρ(r,t) (given by Equation (50)) is assumed to be TD noninteracting ensemble *v*-representable (TD-NI-E-VR) as it belongs to a TD noninteracting ensemble (described by a TD density operator; e.g., see Equation (35)) in the presence of a TD local potential (i.e., the RT-TAO potential).

In RT-TAO-DFT, since we specify the initial state $|\Psi \left(t_{0}\right)\rangle =|\Psi_{\mathrm{GS}}\rangle$ of the physical system and the initial density operator ${\hat{\Gamma }}_{s}\left(t_{0}\right)={\hat{\Gamma }}_{\mathrm{TAO}}$ of the RT-TAO reference system, the two conditions (i) the same initial density $\rho \left(r,t_{0}\right)=\rho_{s}\left(r,t_{0}\right)$ and (ii) the same initial time derivative of the density $\frac{\partial }{\partial t}\rho \left(r,t\right){|}_{t=t_{0}}=\frac{\partial }{\partial t}\rho_{s}\left(r,t\right){|}_{t=t_{0}}=0$ can be satisfied for the physical and RT-TAO reference systems, providing that the GS density $\rho_{\mathrm{GS}}\left(r\right)$ of the unperturbed physical system is NI-TE-VR with a given value of θ (see Equation (38)). Note that conditions (i) and (ii), which ensure the existence of TD-NI-PS-VR densities [86], may also be the conditions for the existence of TD-NI-E-VR densities [87].

In particular, condition (i) highlights the significance of the initial density representability or the GS density representability (for most TD cases of physical interest, the initial state is chosen as the GS of the unperturbed physical system). For an MR system, condition (i) can be violated with conventional TD-DFT since the corresponding GS theory, i.e., KS-DFT, can suffer from the aforementioned issues related to the GS density representability [66,67,68,69,70,71] and the spin-symmetry constraint [3,4,26,83]. By contrast, these issues can be greatly resolved by TAO-DFT (i.e., the underlying GS theory of RT-TAO-DFT) [26,27,28,30,33,38,39,40,41] when an appropriate θ is chosen.

### 3.2. Matrix Representation

In RT-TAO-DFT, the *j*-th RT-TAO orbital $\phi_{j}\left(r,t\right)$ can be expanded in the orthonormal one-electron basis $\left\{\phi_{p}^{0}\left(r\right)\right\}$, spanned by the GS TAO orbitals (i.e., the TAO orbitals associated with the GS of the unperturbed physical system) [26]:

(51) $$\phi_{j}\left(r,t\right)=\sum_{p}C_{pj}\left(t\right)\phi_{p}^{0}\left(r\right),$$

where $\left\{C_{pj}\left(t\right)\right\}$ are the TD expansion coefficients. Accordingly, the TD density ρ(r,t) (see Equation (50)) can be expressed as

(52) $$\rho \left(r,t\right)=\sum_{pq}P_{pq}\left(t\right)\phi_{p}^{0}\left(r\right)\phi_{q}^{0*}\left(r\right),$$

where P(t) is the one-electron density matrix at time *t*, with matrix elements

(53) $$P_{pq}\left(t\right)=\sum_{j}f_{j}C_{pj}\left(t\right)C_{qj}^{*}\left(t\right).$$

Moreover, at time *t*, F(t) is the RT-TAO matrix (commonly known as the Fock matrix), which is the matrix representation of the RT-TAO effective one-electron Hamiltonian ${\hat{h}}_{s}\left(r,t\right)$ (see Equation (47)), with matrix elements

(54) $$F_{pq}\left(t\right)=\int dr\;\phi_{p}^{0*}\left(r\right){\hat{h}}_{s}\left(r,t\right)\phi_{q}^{0}\left(r\right).$$

In the orthonormal one-electron basis $\left\{\phi_{p}^{0}\left(r\right)\right\}$, the RT-TAO equation (given by Equation (48)) can be reformulated in terms of the TD one-electron density matrix P(t) [8]:

(55) $$i\frac{d}{dt}P\left(t\right)=\left[F\left(t\right),P\left(t\right)\right].$$

As the time propagation is assumed to start from the GS of the unperturbed physical system at time t=0 (without loss of generality, the initial time $t_{0}\equiv 0$ is assigned hereafter), the initial one-electron density matrix is given by

(56) $$P_{pq}\left(0\right)=f_{p}\delta_{pq},$$

and the initial RT-TAO matrix is given by

(57) $$F_{pq}\left(0\right)=\epsilon_{p}\delta_{pq},$$

where $f_{p}$ and $\epsilon_{p}$ are the occupation number and energy, respectively, of the *p*-th GS TAO orbital $\phi_{p}^{0}\left(r\right)$ [26].

The formal solution of RT-TAO equation (see Equation (55)) for the TD one-electron density matrix P(t) is given by [60,88,89]

(58) $$P\left(t\right)=U\left(t,0\right)P\left(0\right)U^{†}\left(t,0\right).$$

Here, $U(t_{b},t_{a})$ is a unitary time propagator from $t_{a}$ to $t_{b}$:

(59) $$U\left(t_{b},t_{a}\right)=\hat{T}\exp\left[-i\int_{t_{a}}^{t_{b}}dt^{\prime }\;F\left(t^{\prime }\right)\right],$$

where $\hat{T}$ denotes time ordering. However, since $F(t_{a})$ does not necessarily commute with $F(t_{b})$ for $t_{a}\neq t_{b}$ (see Equation (59)), it remains challenging to obtain P(t) directly from P(0) (see Equation (58)) for a long time interval [0,t] in RT-TAO-DFT.

In practical calculations, to reduce the error in the propagation for a long time interval [0,t], U(t,0) is commonly split into a product of multiple time propagators, each corresponding to a small time step Δ*t*:

(60) $$U\left(t,0\right)=\prod_{n=0}^{m-1}U\left(t_{n+1},t_{n}\right),$$

where $t_{n}=n\Delta t$ denotes the value of *t* at the *n*-th time step, noting that $t_{0}=0$ and $t_{m}=m\Delta t=t$. $U(t_{n+1},t_{n})$ is the time propagator from $t_{n}$ to $t_{n+1}=t_{n}+\Delta t$, given by

(61) $$U\left(t_{n+1},t_{n}\right)=\hat{T}\exp\left[-i\int_{t_{n}}^{t_{n}+\Delta t}dt^{\prime }\;F\left(t^{\prime }\right)\right],$$

which takes $P(t_{n})$ to $P(t_{n+1})$:

(62) $$P\left(t_{n+1}\right)=U\left(t_{n+1},t_{n}\right)P\left(t_{n}\right)U^{†}\left(t_{n+1},t_{n}\right).$$

We denote $U_{n}=U\left(t_{n+1},t_{n}\right)$ and $P_{n}=P\left(t_{n}\right)$ for brevity and apply Equation (60) to Equation (58). Accordingly, the density matrix $P_{m}=P\left(t_{m}\right)=P\left(t\right)$ can be obtained from the initial density matrix $P_{0}=P\left(t_{0}\right)=P\left(0\right)$ via the following expression:

(63) $$\begin{array}{cc} P_{m}= & \;\left(\prod_{n=0}^{m-1}U_{n}\right)P_{0}{\left(\prod_{n=0}^{m-1}U_{n}\right)}^{†}=U_{m-1}U_{m-2}\cdots U_{1}U_{0}P_{0}U_{0}^{†}U_{1}^{†}\cdots U_{m-2}^{†}U_{m-1}^{†}. \end{array}$$

For a sufficiently small time step Δ*t*, $F(t_{n})$ remains nearly commutative with $F(t_{n}+\Delta t)$, and, hence, $U_{n}=U\left(t_{n+1},t_{n}\right)$ (see Equation (61)) can be computed without considering the time ordering. Note, however, that the exact time-ordered propagator can only be obtained in the limit of an infinitesimal time step (i.e., Δ*t*→0).

Recently, various algorithms [60,88,89] have been developed for the numerical construction of the time propagation of TDKS equation [6], which may also be adopted for the time propagation of RT-TAO equation.

In short, it takes the following key steps to run an RT-TAO-DFT calculation for describing the time evolution of the electron density following a perturbation:

- Construct the initial one-electron density matrix P(0) (see Equation (56)) and the initial RT-TAO matrix F(0) (see Equation (57)) for the GS of the unperturbed physical system at time t=0 using TAO-DFT (i.e., the respective GS theory).
- Apply the TD field to the physical system for t>0, and propagate the one-electron density matrix P(t) and the RT-TAO matrix F(t) in the time domain, according to the RT-TAO equation (given by the matrix representation, e.g., see Equation (55)).
- Post-process the resulting TD observables (electron density, dipole moment, etc.).

## 4. HHG Spectra from RT-TAO-DFT

HHG from an electronic system (e.g., an atom or molecule) is a nonlinear optical process driven by an intense laser pulse, wherein the laser frequency can be converted into its integer multiples [43,44,45,46,47,48,49,50,51,52,53,54,55,56,57,58,59,60]. HHG has recently attracted much attention since it can be used to explore the structure and dynamics of electronic systems and chemical reactions on a femtosecond timescale. In addition, HHG can be employed to generate attosecond pulse trains as well as individual attosecond pulses [45,46].

HHG can be qualitatively described by the semiclassical three-step model [43,44]. First, an electron tunnels out from an electronic system in an intense laser field (i.e., tunnel ionization). Second, the electron is driven away from or back to the parent ion by the laser field. Finally, the electron recombines with the parent ion, emitting a high-energy photon.

In this work, we perform RT-TAO-DFT calculations to explicitly obtain the HHG spectra and related TD properties of molecular hydrogen $H_{2}$ at the equilibrium and stretched geometries:

- $H_{2}$ with an equilibrium bond length of 1.45 bohr (≈0.767 Å).
- $H_{2}$ with a stretched bond length of 3.78 bohr (≈2.00 Å).

Here, the nuclei of $H_{2}$ are positioned along the *z*-axis (i.e., the laser polarization) with the center of mass being located at the origin.

**Figure 1:**
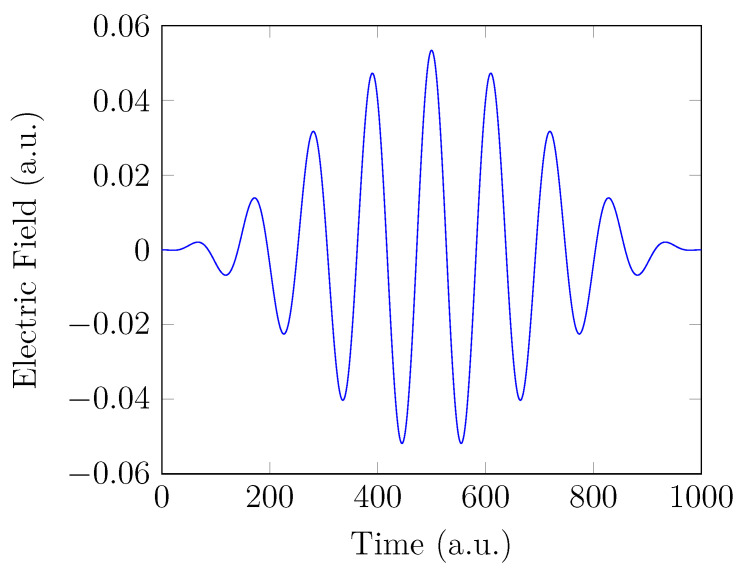
Electric field of the laser pulse adopted.

To obtain the HHG spectrum, the electronic system $H_{2}$, which starts from the GS at time t=0, experiences an intense laser pulse for t>0. In order to mimic the commonly used Ti:sapphire laser [90], the strong-field interaction is generated by a laser pulse with an oscillating electric field linearly polarized along the *z*-axis (see Figure 1):

(64) $$v_{\mathrm{laser}}\left(r,t\right)=zA_{0}\cos^{2}\left[\frac{\pi }{2\sigma_{p}}\left(t-\sigma_{p}\right)\right]\cos\left[\omega_{0}\left(t-\sigma_{p}\right)\right].$$

Here, the interaction with the electric field is treated in the dipole approximation and the length gauge [91]. The electric-field amplitude of the laser pulse $A_{0}$ = 0.0534 a.u. (corresponding to the peak intensity $I_{0}\approx 1\times 10^{14}$ W/cm^2^), the laser frequency (also called the fundamental frequency) $\omega_{0}$ = 1.5498 eV (corresponding to the wavelength $\lambda_{0}\approx$ 800 nm), and $\sigma_{p}$ = 500 a.u. (≈12.1 fs) are adopted.

In the HHG process, the electron released by tunnel ionization can travel far away from the center of the electronic system $H_{2}$. To capture strong-field ionization process and to remove artificial reflections induced by the finite extent of Gaussian basis set (which will be adopted to describe the TAO/RT-TAO orbitals), a complex absorbing potential (CAP) [60], $-iv_{\mathrm{CAP}}\left(r\right)$, is also employed for t>0. For an electronic system consisting of $N_{A}$ atoms, the CAP function $v_{\mathrm{CAP}}\left(r\right)$ is defined as the minimum of the values of the atom-centered spherical absorbing potentials:

(65) $$v_{\mathrm{CAP}}\left(r\right)=\min\left\{g_{1}\left(r\right),...,g_{N_{A}}\left(r\right)\right\}.$$

Here, $g_{I}\left(r\right)$ is a spherical absorbing potential around the *I*-th nucleus:

(66) $$g_{I}\left(r\right)=\left\{\begin{array}{ccc} 0, & for & \left|r-R_{I}\right|<r_{0}\\ \eta {\left(\left|r-R_{I}\right|-r_{0}\right)}^{2}, & for & r_{0}\leq \left|r-R_{I}\right|<r_{0}+\sqrt{V_{\max}/\eta }\\ V_{\max}, & for & r_{0}+\sqrt{V_{\max}/\eta }\leq \left|r-R_{I}\right| \end{array}\right.$$

for *I* = 1, …, $N_{A}$, where $R_{I}$ is the position of the *I*-th nucleus. The cutoff radius $r_{0}$ should be small enough to interact with the electron density (because the space extended by the Gaussian basis set is finite), while it should be large enough to not overly perturb the original electronic system. Here, we adopt the cutoff radius $r_{0}$ = 9.524 bohr (≈5.040 Å), the curvature η = 4.0 hartree/bohr^2^, and the maximum potential value *V*_max_ = 10 hartree.

To sum up, in the present HHG study, the RT-TAO effective one-electron Hamiltonian ${\hat{h}}_{s}\left(r,t\right)$ (see Equation (47)) is given by

(67) $${\hat{h}}_{s}\left(r,t\right)=\left\{\begin{array}{ccc} {\hat{h}}_{s}^{0}\left(r,t\right), & for & t\leq 0\\ {\hat{h}}_{s}^{0}\left(r,t\right)+v_{\mathrm{laser}}\left(r,t\right)-iv_{\mathrm{CAP}}\left(r\right), & for & t>0 \end{array}\right.$$

where ${\hat{h}}_{s}^{0}\left(r,t\right)$ is the field-free RT-TAO effective one-electron Hamiltonian, $v_{\mathrm{laser}}\left(r,t\right)$ (see Equation (64)) is the strong-field interaction, and $-iv_{\mathrm{CAP}}\left(r\right)$ (see Equation (65)) is the CAP.

To propagate the one-electron density matrix P(t) in the time domain, we adopt a time step of Δ*t* = 0.02 a.u. (≈ 0.484 as) and a total propagation time of τ = 1000 a.u. (≈24.1 fs), which corresponds to a total of $5\times 10^{4}$ time steps. The modified mid-point unitary transformation (MMUT) algorithm [88,89] is employed for the numerical time propagation of P(t). As the use of the CAP breaks the conservation of the norm of the RT-TAO orbitals, the time propagation is no longer unitary [60].

Following sufficient time propagation, the TD one-electron density matrix P(t) is determined, and the TD density ρ(r,t) is given by Equation (52), yielding various TD properties. While the RT-TAO orbital occupation numbers, which are the same as the GS TOONs $\left\{f_{j}\right\}$, are time-independent, the norm of the RT-TAO orbitals can decrease with time *t* due to the CAP. To describe electron ionization, the number of bound electrons is computed using

(68) $$N_{b}\left(t\right)=\int dr\;\rho \left(r,t\right)=\int dr\;\sum_{j}f_{j}{\left|\phi_{j}\left(r,t\right)\right|}^{2}=\mathrm{Tr}\left\{P\left(t\right)\right\}.$$

Moreover, the induced dipole moment along the laser polarization (i.e., the *z*-axis) is calculated by

(69) μ(t)=−∫drzρ(r,t).

Accordingly, the HHG spectrum can be computed using [9,92]

(70) $$H\left(\omega \right)=\frac{1}{2\pi }{\left|\int_{0}^{\tau }dt\;w_{H}\left(t\right)\frac{d^{2}\mu \left(t\right)}{dt^{2}}e^{-i\omega t}\right|}^{2},$$

where the HHG spectrum has been smoothed using the Hamming window function

(71) $$w_{H}\left(t\right)=0.54-0.46\cos\left(\frac{2\pi t}{\tau }\right)$$

to reduce the numerical noise. In the HHG spectrum, the harmonic order is defined as $\omega /\omega_{0}$, with $\omega_{0}$ being the fundamental frequency (see Equation (64)).

Here, we present the approximations made in the RT-TAO-DFT calculations, the computational details, and the numerical results. As the exact TD xc*θ* potential $v_{\mathrm{xc}\theta }\left(r,t\right)=\frac{\delta A_{\mathrm{xc}\theta }\left[\rho \right]}{\delta \rho (r,t)}$ (see Equation (46)) remains unknown, approximations to $v_{\mathrm{xc}\theta }\left(r,t\right)$ are necessary for practical RT-TAO-DFT calculations. While the exact $v_{\mathrm{xc}\theta }\left(r,t\right)$ formally depends on the density $\rho (r,t^{\prime })$ at all previous times $t^{\prime }\leq t$, in this study, we make the adiabatic approximation:

(72) $$v_{\mathrm{xc}\theta }\left(r,t\right)\approx \frac{\delta E_{\mathrm{xc}\theta }\left[\rho \right]}{\delta \rho (r)}{|}_{\rho (r)=\rho (r,t)},$$

where the TD xc*θ* potential $v_{\mathrm{xc}\theta }\left(r,t\right)$ is approximated by the GS xc*θ* potential $\frac{\delta E_{\mathrm{xc}\theta }\left[\rho \right]}{\delta \rho (r)}$ (see Equation (6)) evaluated at the instantaneous density ρ(r,t). In the adiabatic approximation, since the exact xc*θ* energy functional $E_{\mathrm{xc}\theta }\left[\rho \right]$ also remains unknown, a DFA to $E_{\mathrm{xc}\theta }\left[\rho \right]$ should be made as well. In this work, we adopt the LDA (i.e., the simplest DFA) xc*θ* energy functional $E_{\mathrm{xc}\theta }^{\mathrm{LDA}}\left[\rho \right]=E_{\mathrm{xc}}^{\mathrm{LDA}}\left[\rho \right]+E_{\theta }^{\mathrm{LDA}}\left[\rho \right]$, with $E_{\mathrm{xc}}^{\mathrm{LDA}}\left[\rho \right]$ being the LDA xc energy functional [11,12] and $E_{\theta }^{\mathrm{LDA}}\left[\rho \right]$ being the LDA θ-dependent energy functional [26]. For brevity, RT-TAO-DFT with the adiabatic LDA xc*θ* potential is denoted as RT-TAO-ALDA. At time t=0, the initial state (i.e., the GS of the unperturbed $H_{2}$ with a given bond length) is obtained with the underlying GS theory, TAO-LDA (i.e., TAO-DFT with the LDA xc*θ* energy functional $E_{\mathrm{xc}\theta }^{\mathrm{LDA}}\left[\rho \right]$) [26]. Note that RT-TAO-ALDA (with θ=0) corresponds to TD-ALDA (i.e., conventional TD-DFT with the adiabatic LDA xc potential) [7,8,9], and its underlying GS theory, TAO-LDA (with θ=0), corresponds to KS-LDA (i.e., KS-DFT with the LDA xc energy functional $E_{\mathrm{xc}}^{\mathrm{LDA}}\left[\rho \right]$) [11,12].

In this study, we also investigate the possible spin-symmetry breaking effects in the TD properties, in analogy to the GS counterparts [3,4,26,83]. At time t=0, the initial state (i.e., the GS of the unperturbed $H_{2}$ with a given bond length) is a singlet state, and, hence, the initial up-spin and down-spin densities obtained with an exact theory must be the same based on the spin-symmetry constraint [3,4,26,83]. Moreover, for t>0, since the TD external potential adopted is spin-independent (see Equation (67)), the up-spin and down-spin densities, which are equally propagated in the time domain, must be the same at any subsequent time *t* [93]. Therefore, the TD properties (which depend on the TD spin densities) of $H_{2}$ obtained with the spin-unrestricted formalism must be identical to those obtained with the spin-restricted formalism.

To examine whether this spin-symmetry constraint can be satisfied by RT-TAO-ALDA, we perform spin-restricted and spin-unrestricted RT-TAO-ALDA (with θ = 0, 7, 20, and 40 mhartree) calculations for the TD properties, such as the number of bound electrons, induced dipole moment, and HHG spectrum, of $H_{2}$ at the equilibrium and stretched geometries (aligned along the polarization of an intense linearly polarized laser pulse) using the d-aug-cc-pVTZ basis set and a high-quality grid EML(99,590) containing 99 Euler–Maclaurin radial grid points and 590 Lebedev angular grid points. We note that the choice of basis set can significantly affect the HHG spectrum [94]. For the special case of θ=0, RT-TAO-ALDA reduces to TD-ALDA. All numerical results are obtained with a development version of Q-Chem 5.4 [95].

**Figure 2:**
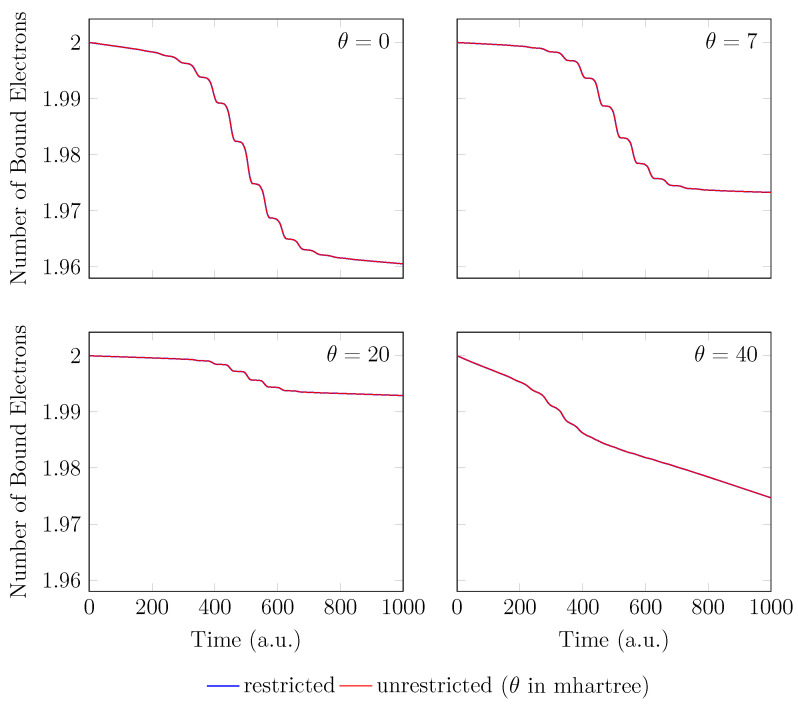
Number of bound electrons for $H_{2}$ with an equilibrium bond length of 1.45 bohr, obtained with spin-restricted and spin-unrestricted RT-TAO-ALDA (with various θ). Here, the θ=0 case corresponds to TD-ALDA.

**Figure 3:**
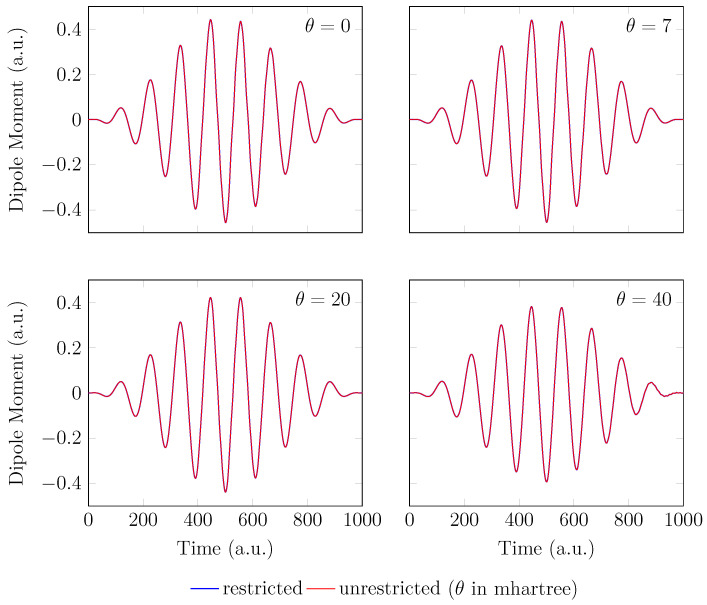
Induced dipole moment for $H_{2}$ with an equilibrium bond length of 1.45 bohr, obtained with spin-restricted and spin-unrestricted RT-TAO-ALDA (with various θ). Here, the θ=0 case corresponds to TD-ALDA.

**Figure 4:**
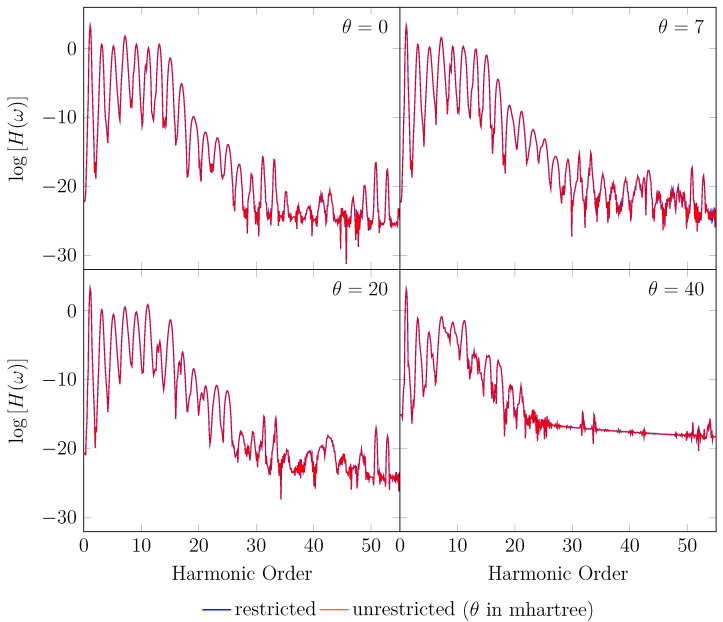
HHG spectrum for $H_{2}$ with an equilibrium bond length of 1.45 bohr, obtained with spin-restricted and spin-unrestricted RT-TAO-ALDA (with various θ). Here, the θ=0 case corresponds to TD-ALDA.

Since the GS of the unperturbed $H_{2}$ with an equilibrium bond length of 1.45 bohr exhibits mainly SR character, the spin-symmetry constraint can be satisfied by spin-unrestricted TAO-LDA (with θ = 0, 7, 20, and 40 mhartree) [26], producing the same up-spin and down-spin densities at time t=0. In addition, for t>0, owing to the use of a TD spin-independent external potential, the up-spin and down-spin densities, which are equally propagated in the time domain, should be the same at any subsequent time *t* [93]. Therefore, the TD properties, such as the number of bound electrons (see Figure 2), induced dipole moment (see Figure 3), and HHG spectrum (see Figure 4), of $H_{2}$ with an equilibrium bond length of 1.45 bohr, obtained with spin-restricted and spin-unrestricted RT-TAO-ALDA (with θ = 0, 7, 20, and 40 mhartree), are essentially the same (i.e., within the numerical precision considered).

**Figure 5:**
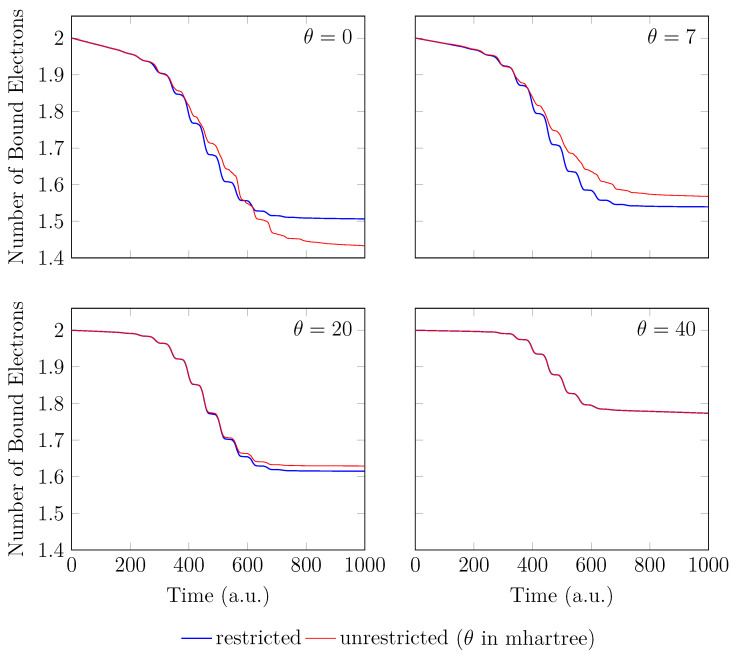
Number of bound electrons for $H_{2}$ with a stretched bond length of 3.78 bohr, obtained with spin-restricted and spin-unrestricted RT-TAO-ALDA (with various θ). Here, the θ=0 case corresponds to TD-ALDA.

**Figure 6:**
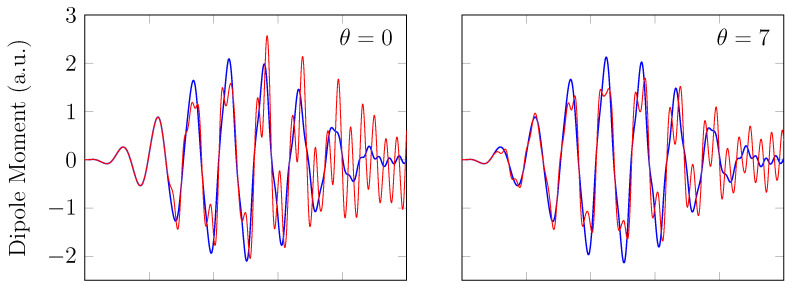

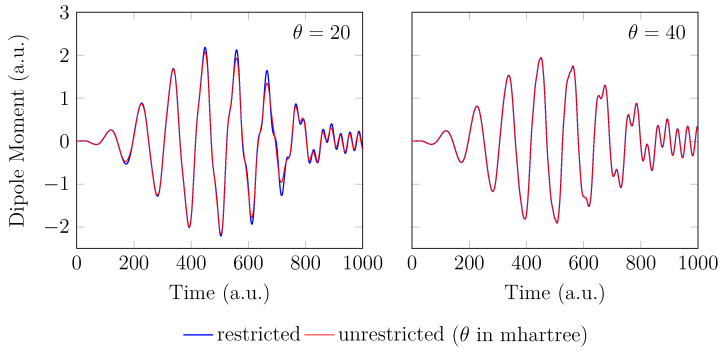
Induced dipole moment for $H_{2}$ with a stretched bond length of 3.78 bohr, obtained with spin-restricted and spin-unrestricted RT-TAO-ALDA (with various θ). Here, the θ=0 case corresponds to TD-ALDA.

**Figure 7:**
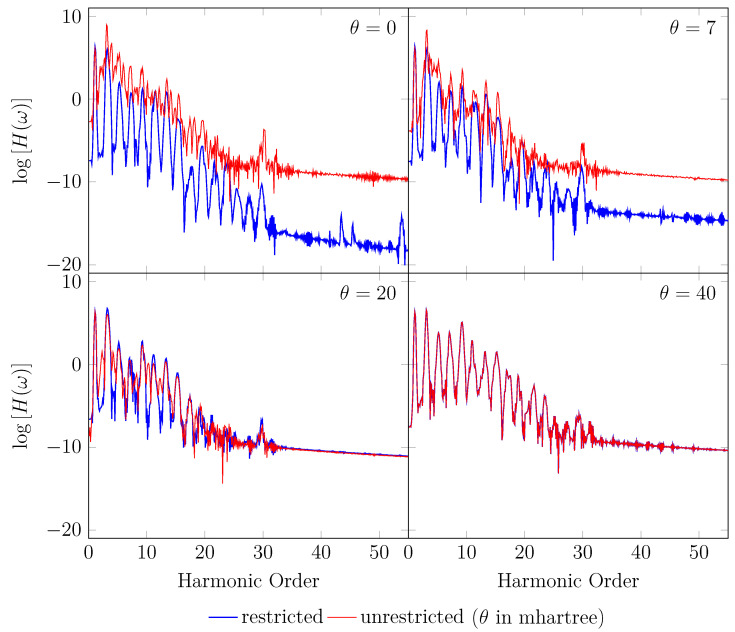
HHG spectrum for $H_{2}$ with a stretched bond length of 3.78 bohr, obtained with spin-restricted and spin-unrestricted RT-TAO-ALDA (with various θ). Here, the θ=0 case corresponds to TD-ALDA.

On the other hand, the GS of the unperturbed $H_{2}$ with a stretched bond length of 3.78 bohr exhibits a noticeable MR character [26], and, hence, the spin-symmetry constraint is violated with spin-unrestricted KS-LDA (i.e., TAO-LDA with θ=0), producing symmetry-broken spin densities at time t=0. In this situation, even when a TD spin-independent external potential is applied to the initial state (i.e., a spin-symmetry-broken GS) for t>0, the TD effective one-electron potentials can be spin-dependent, and, hence, the up-spin and down-spin densities, which are unequally propagated in the time domain, can be very different at any subsequent time *t*. As shown, the TD properties, such as the number of bound electrons (see Figure 5), induced dipole moment (see Figure 6), and HHG spectrum (see Figure 7), of H_2_ with a stretched bond length of 3.78 bohr, obtained with spin-restricted and spin-unrestricted TD-ALDA (i.e., RT-TAO-ALDA with *θ* = 0), are distinctly different, yielding unphysical spin-symmetry breaking effects in all the TD properties examined. Such an unphysical spin-symmetry breaking feature of spin-unrestricted TD-ALDA is apparently undesirable for RT simulations. By contrast, the spin-symmetry breaking effects in the TD properties obtained with RT-TAO-ALDA are shown to be reducible with the increase in *θ* at essentially no additional computational cost. In particular, the TD properties obtained with spin-restricted and spin-unrestricted RT-TAO-ALDA (with *θ* = 40 mhartree) are essentially the same, yielding essentially no unphysical spin-symmetry breaking effects in all the TD properties examined. This desirable feature can be attributed to the satisfaction of spin-symmetry constraint on the singlet GS density of the stretched H_2_ by spin-unrestricted TAO-LDA (with *θ* = 40 mhartree) [26].

## 5. Conclusions

In conclusion, we have developed RT-TAO-DFT (i.e., an RT extension of TAO-DFT [26]), allowing the study of TD properties of both SR and MR systems. By resorting to an ensemble formalism, RT-TAO-DFT has resolved the aforementioned inconsistency of TDTAO-DFT (especially for θ≠0) [42]. Since the assumption of a weak perturbation is not required in RT-TAO-DFT, spin-restricted and spin-unrestricted RT-TAO-DFT (with various θ) calculations have been performed to explore the TD properties (e.g., the number of bound electrons, induced dipole moment, and HHG spectrum) of $H_{2}$ at the equilibrium and stretched geometries, aligned along the polarization of an intense linearly polarized laser pulse. The TD properties obtained with RT-TAO-DFT (with various θ) have been compared with those obtained with conventional TD-DFT [6], which corresponds to RT-TAO-DFT (with θ=0). Moreover, issues related to the possible spin-symmetry breaking effects in the TD properties are also discussed.

## Figures and Tables

**Table 1:** Comparison of KS-DFT [1], TAO-DFT [26], and FT-DFT [1,62].

	KS-DFT	TAO-DFT	FT-DFT
Electronic Temperature $\theta_{el}$	0	0	≥0
Fictitious Temperature θ	0	≥0	≥0
Is $\theta =\theta_{el}$ assumed?	Yes	No	Yes
Electronic Property	GS	GS	Thermal Equilibrium
Electron Density	GS	GS	Thermal Equilibrium
Density Representation	NI-PS-VR	NI-TE-VR	NI-TE-VR
Universal Functional	Hohenberg–Kohn	Hohenberg–Kohn	Mermin
Approximate Functional	$E_{\mathrm{xc}}\left[\rho \right]$	$E_{\mathrm{xc}\theta }\left[\rho \right]$	$F_{\mathrm{xc}}^{\theta_{el}}\left[\rho^{\theta_{el}}\right]$

**Table 2:** Comparison of TAO-DFT (with $E_{\mathrm{xc}\theta }\left[\rho \right]\approx E_{\mathrm{xc}}\left[\rho \right]$) [26] and FT-DFT (with $F_{\mathrm{xc}}^{\theta_{el}}\left[\rho^{\theta_{el}}\right]\approx E_{\mathrm{xc}}\left[\rho^{\theta_{el}}\right]$) [1,62].

	TAO-DFT (with $E_{\mathrm{xc}\theta }\left[\rho \right]\approx E_{\mathrm{xc}}\left[\rho \right]$)	FT-DFT (with $F_{\mathrm{xc}}^{\theta_{\mathrm{el}}}\left[\rho^{\theta_{\mathrm{el}}}\right]\approx E_{\mathrm{xc}}\left[\rho^{\theta_{\mathrm{el}}}\right]$)
Electronic Temperature $\theta_{el}$	0	≥0
Fictitious Temperature θ	≥0	≥0
Is $\theta =\theta_{el}$ assumed?	No	Yes
Electronic Property	GS	Thermal Equilibrium
Electron Density	GS	Thermal Equilibrium
Density Representation	NI-TE-VR	NI-TE-VR
Approximate Functional	$E_{\mathrm{xc}}\left[\rho \right]$	$E_{\mathrm{xc}}\left[\rho^{\theta_{el}}\right]$

## Data Availability

The numerical data supporting the findings of the present work are available from the authors upon appropriate request.
